# Comment from the Editor to the Special Issue: “Big Data and Precision Medicine Series I: Lung Cancer Early Diagnosis”

**DOI:** 10.3390/jcm7020028

**Published:** 2018-02-09

**Authors:** Roberto Gasparri, Giulia Sedda, Lorenzo Spaggiari

**Affiliations:** 1 Department of Thoracic Surgery, European Institute of Oncology, 20141 Milan, Italy; giulia.sedda@ieo.it (G.S.); lorenzo.spaggiari@ieo.it (L.S.); 2 Department of Oncology and Emato-Oncology, University of Milan, 20122 Milan, Italy

**Keywords:** big data, lung cancer, early diagnosis

## Abstract

With this *Editorial* we want to present the Special Issue “Big Data and Precision Medicine Series I: Lung Cancer Early Diagnosis” to the scientific community, which aims to gather experts on the early detection of lung cancer in order to implement common efforts in the fight against cancer.

Recent years have borne witness to the emergence of a new approach to cancer diagnosis. The focus has shifted from the classical idea of “the tumor as a single entity” towards that of metabolism, its changes, and its products. Even though cancer in its early stages, on which the diagnostic approach must be centered, is still too small to be detected, several techniques have shown considerable promise in detecting metabolic changes [1]. The improvement of technology such as next-generation sequencing and the other “omics” techniques have driven a novel type of medicine focused on the patient’s characteristics and disease features [2]. 

The major challenge of these new techniques is the large heterogeneous amount of data generated from the analysis of a single patient concerning, for instance, genetic expression, proteomics, or metabolic analysis. This data has been estimated to reach the petabyte range (i.e., 1000 terabytes) for each analysis. The information needs to be gathered, processed, compared, and analyzed in order to find out whether it can be useful for a synergistic approach to an early diagnosis of the disease. 

Together with the increased quantity of data obtainable through different studies, we must also bear in mind the importance of its quality: much of the information could represent bias or background noise that needs to be corrected for or filtered out. Furthermore, in parallel with the improvement of the techniques and the amount of data they yield, the methodological data generated from individual subject studies have grown to reach huge levels. For instance, the classical paper-based manual medical record has undergone a transformation into a detailed electronic medical record—an EMR—with the contribution of the different specialists, thereby allowing a multidisciplinary and effective approach. As suggested by Williams and colleagues, there is the need to translate such big data into clinically useful knowledge [3]. 

The results obtained by the many and varied techniques (such as medical history analysis, genomics, proteomics, or metabolomics) could allow us to stratify the patient with a collective approach in an ideal scenario of a highly sensitive and specific screening algorithm. This would result in an improvement of diagnosis and, most importantly, allow the patient a better chance of survival. 

Furthermore, considering the widespread diffusion of multicenter studies and clinical trials, there is the pressing need to disseminate readily accessible information around the world. The sharing of big data, essential to guarantee progress in the field of early diagnosis, is a crucial step in diagnostic implementation. 

For other types of tumors, these systematic analyses and information sharing methods have already been approved. For instance, in 2016, several nations worldwide, including Australia, Canada, Japan, Sweden, the United Kingdom, and the United States, collaborated to create the International Cancer Proteogenome Consortium [4]. This Consortium aims to coordinate efforts in the fight against cancer and bring together different scientists to foster new insights into cancer and improve its treatment and survival. The development of a world network focused on the screening approach would allow big biological data arising from lung cancer research to be translated into clinical practice, so that each piece of the information jigsaw can contribute to solving the larger puzzle of early diagnosis.
